# Silk Fibroin Hydrogel Microneedles Loaded with Recombinant Human Nerve Growth Factor for Corneal Tissue Engineering

**DOI:** 10.3390/polym18030412

**Published:** 2026-02-05

**Authors:** Jinmei Zhang, Linran Song, Xinrang Zhai, Dilnaz Em, Xihao Pan

**Affiliations:** 1 Department of Ophthalmology, Center for Regeneration and Aging Medicine, The Fourth Affiliated Hospital of School of Medicine, and International Institutes of Medicine, Zhejiang University, Yiwu 322000, China; zhangmeimei6@zju.edu.cn (J.Z.); 18335751799@163.com (L.S.); zxrmsxyy@163.com (X.Z.);; 2 School of Chemistry and Chemical Engineering, Nanjing University of Science and Technology, Nanjing 210094, China; 3 Liangzhu Laboratory, Zhejiang University School of Medicine, Hangzhou 310003, China

**Keywords:** hydrogel, microneedles, recombinant human nerve growth factor, drug delivery, corneal tissue engineering

## Abstract

Corneal nerves are essential for maintaining the functional integrity of the ocular surface. Damage to corneal nerves can lead to corneal issues and impaired vision. Current treatments for corneal nerve damage are inadequate, thus highlighting the need for innovative therapeutic approaches. In this study, we present a hydrogel microneedle system designed to facilitate the sustained release of recombinant human nerve growth factor (rhNGF). The microneedle features a tip composed of glycidyl methacrylate modified silk fibroin (SFMA) loaded with rhNGF, photopolymerized for structural integrity, while its base is formed using silk fibroin (SF). This design allows the microneedles to penetrate the corneal epithelium and deliver rhNGF to the sub-epithelial layer. The crosslinking process not only provides the mechanical strength required for microneedle penetration but also enables sustained drug release. The proposed rhNGF-loaded SF hydrogel microneedle provides a platform for drug delivery, serving as a novel therapeutic option for corneal tissue engineering.

## 1. Introduction

The cornea, an avascular yet heavily innervated anterior ocular tissue, plays a critical role in protecting intraocular structures and shielding them from external environmental damage and pathogens [1,2,3,4,5]. Corneal nerves are essential for maintaining ocular surface homeostasis [6,7]. However, corneal nerves are vulnerable to various insults, such as dry eye syndrome, infections, chemical injuries, and diabetes, which can lead to vision impairment and reduced quality of life [8]. Current therapeutic approaches include topical eye drops and corneal neurotization surgery [9,10]. Topical eye drops are convenient to administer. However, the unique anatomy and physiology of the cornea limit drug absorption. Specifically, the tight junctions of epithelial cells form a barrier that impedes drug penetration [11,12]. These limitations result in short drug efficacy durations, low bioavailability, and potential drug degradation on the ocular surface, thereby impeding nerve healing [13]. Additionally, the requirement for long-term use of eye drops places a significant burden on patient compliance, which may compromise therapeutic outcomes if usage frequency and duration are not adhered to [14]. On the other hand, surgical interventions may address the root cause of nerve damage and help maintain corneal transparency and nerve function over time. However, such procedures are invasive and may lead to greater tissue trauma, such as corneal defects or corneal and conjunctival scarring [15]. These complications can further hinder nerve repair [16]. Furthermore, surgical success depends heavily on the surgeon’s expertise. Given these challenges, there is an urgent need to develop novel therapeutic strategies for corneal nerve injury that are minimally invasive, long-lasting, and highly effective to improve patient outcomes.

Recombinant human nerve growth factor (rhNGF) is a critical growth factor that plays a pivotal role in regulating neuron survival and maturation, as well as in promoting corneal nerve regeneration [17]. In recent years, rhNGF has gained increasing attention in the medical field due to its remarkable effectiveness in treating moderate-to-severe neurotrophic keratopathy, further demonstrating its safety and efficacy [18]. However, conventional eye drops are associated with a bioavailability of less than 5% [19,20]. Microneedle technology has emerged as an innovative drug delivery approach that bypasses the corneal epithelial barrier to directly deliver drugs to target tissues, enabling pain-free, non-invasive, and efficient drug delivery [21,22,23]. Microneedles protect the drug within a controlled-release matrix, reducing its degradation and enabling a sustained drug release profile [24]. This not only minimizes dosing frequency but also enhances patient compliance [25]. Within ophthalmic therapy, microneedles can incorporate drugs, functional growth factors, and stem cells, thereby expanding therapeutic opportunities and offering a new dimension to the treatment of ocular diseases [26]. This innovation has significantly propelled the advancement of ophthalmic therapeutic technologies. Recent years have seen a growing focus in the field of corneal repair on silk fibroin (SF) and its derivatives, with accumulating evidence underscoring their safety and efficacy [27,28]. Gong et al. developed a novel targeted drug against the substance P/neurokinin-1 receptor (SP/NK1R) pathway. They utilized SF-loaded NK1R antagonists (CP99,994) to construct a nanoparticle-based drug delivery system, which was further integrated with a hydrogel for ocular drug delivery. This innovative approach offers a promising solution for the treatment of dry eye disease [29]. Meng et al. developed a multifunctional hybrid hydrogel composed of glycidyl methacrylate modified silk fibroin (SFMA) and chitosan using a double-network strategy. This hydrogel is designed to repair corneal wounds with severe bacterial infections and possesses spatiotemporal drug release characteristics [30]. RhNGF and SFMA have been extensively developed and widely applied in their respective fields. However, the composite system combining these two components has been scarcely investigated for corneal nerve repair applications.

Herein, we developed an innovative delivery system for rhNGF-loaded SFMA microneedles, which combines rhNGF and SFMA needles with an SF base (MN-rhNGF). Microneedles represent an innovative corneal delivery system, once inserted into the cornea, the microneedles hydrate and swell, accurately delivering rhNGF to the corneal tissue for effective drug administration. In addition, the unique material properties of SFMA microneedles enable sustained rhNGF release. This prolongs the retention of rhNGF in the cornea and improves the drug’s utilization efficiency. In vitro biocompatibility assessments were conducted by co-culturing MN-rhNGF extracts at varying concentrations with human corneal epithelial cells (HCECs) and mouse fibroblasts (L929). The results demonstrated good cell compatibility and low cytotoxicity, indicating that the microneedle system holds significant promise in corneal nerve regeneration therapy.

## 2. Materials and Methods

### 2.1. Materials

Na_2_CO_3_ and fluorescein isothiocyanate-labeled bovine serum albumin (FITC-BSA) were purchased from Solarbio (Beijing, China). Glycidyl methacrylate (GMA) was purchased from Sigma-Aldrich (St. Louis, MO, USA). Lithium phenyl-2,4,6-trimethylbenzoylphosphinate (LAP) were purchased from Haining Jurassic Bio-tech company Ltd. Procell Life Science & Technology Co., Ltd. (Wuhan, China) was the vendor of human corneal epithelial cells (HCECs), mouse fibroblasts (L929) cells, and HCEC-specific medium. Low-glucose medium was purchased from Jiangsu KeyGEN Bio TECH Corp., Ltd. (Nanjing, China). Fetal bovine serum (FBS) was purchased from Gibco Life Sciences Company (Grand Island, NY, USA). Penicillin-streptomycin was purchased from Huzhou Cienry Biotechnology Co., Ltd. (Huzhou, China)

### 2.2. Preparation of SF Solution

SF solution was prepared as described previously [31]. Briefly, the raw silk fibers (Bombyx mori, Haiyan County Minghe Silkworm Professional Cooperative, Jiaxing, China) were added to the boiling 0.02 M Na_2_CO_3_ solution for 30 min to separate the sericin proteins from the fibroin proteins. Then, after being rinsed and dried, the extracted fibroin was dissolved into the 9.3 M LiBr solution (Mackin, Shanghai, China) at 60 °C for 4 h. The dissolved SF solution was added to a boiled dialysis bag (molecular weight cut-off: 8000–14,000, Beijing Solarbio Science & Technology Co., Ltd., Beijing, China) and dialyzed against the Milli-Q water for 48 h with six water changes. Finally, the dialyzed SF solution was centrifuged at 9000 rpm at 4 °C twice, and every centrifugation lasted for 20 min. The supernatant was collected and stored at 4 °C after measuring its concentration (6.5% (*w*/*v*)) via dehydration and weighting. RhNGF was purchased from MedChemExpress (Shanghai, China, Storage: −20 °C (stable for 2 years). Upon reconstitution, stable at 4 °C for 1 week or at −20 °C for longer periods (with carrier protein)).

### 2.3. Preparation of SFMA

The preparation process of SFMA involves several steps: silk fibroin (SF) degumming, dissolving SF, reaction with glycidyl methacrylate (GMA), dialysis, freeze-drying, and mixing with LAP. First, cut the silkworm cocoon, remove the inner integument with tweezers, then cut the outer integument into small pieces and put them into boiling 0.02 M Na_2_CO_3_ for 30 min to separate sericin from silk fibers. After washing and drying the silk, degummed silk is obtained. Dissolve the degummed silk in 9.3 M LiBr solution at 60 °C for 4 h. Add GMA and stir at 60 °C for 6 h. After the reaction, put the mixture in a D45 dialysis bag, dialyze against deionized water for 48 h with six water changes. Finally, freeze-dry the solution to get SFMA solid, which is sealed and stored at room temperature.

### 2.4. ^1^H-NMR

SF and SFMA samples were dissolved in deuterium oxide (D_2_O) solution to form a homogeneous solution. The ^1^H nuclear magnetic resonance (^1^H-NMR) spectra were precisely measured using a Bruker Advance III HD 600 MHz NMR spectrometer with experimental parameters set to 16 scans and a spectral width of 20 ppm.

### 2.5. Attenuated Total Reflection Fourier Transform Infrared Spectrometer

Attenuated total reflection Fourier transform infrared spectrometer (ATR-FTIR, Bruker INVENIO S, Billerica, MA, USA) was used to study the molecular characters of SF, SFMA. Before sample analysis, the table and diamond crystal (ATR head) where the sample was placed were cleaned with anhydrous ethanol. After the anhydrous ethanol evaporates, the background spectrum (e.g., H_2_O, CO_2_) was collected and subtracted from the sample spectra. Each sample was scanned 16 times across a spectral range of 2000~500 cm^−1^ at a resolution of 4 cm^−1^.

### 2.6. Preparation of Microneedles

Microneedles were fabricated using the molding technique. First, prepare the rhNGF-loaded SFMA matrix solution. Dissolve SFMA in deionized water at gradient concentrations (5%, 10%, 15%, 20%, *w*/*v*), then quantitatively add rhNGF to a final concentration of 20 μg/mL. Stir thoroughly to form a homogeneous composite system. Add LAP (5%) to form the pre-polymer solution. Next, precisely inject the SFMA/rhNGF mixed solution into the tip micropore array of PDMS, ensuring complete filling (Mold parameters: array density, 20 × 20 (400 needles); inter-needle spacing, 200 μm; base diameter, 250 μm; needle height, 600 μm). To eliminate bubble interference in microneedle morphology introduced during solution filling, place the loaded mold in a vacuum drying apparatus until bubbles are completely removed. Then, gently remove residual solution from the mold surface using a precision scraper, followed by photopolymerization under UV light (365 nm, 100 mW/cm^2^) for 20 s. Subsequently, coat the tips with 6.5% SF solution and dry at 60 °C. Finally, carefully remove the mold to obtain the preliminary microneedle patch, followed by overnight drying at 60 °C.

### 2.7. Characterization of Microneedles

The macroscopic structures and top views of the microneedles were examined using inverted microscopy, while their microscopic structures and top views were analyzed at higher resolution via scanning electron microscopy (SEM, Mira4, Tescan, Brno, Czech Republic).

### 2.8. Hydrogel Transmittance Test

The hydrogel transmittance was determined following a previously described method [32]. To investigate the impact of concentration on the optical properties of hydrogels, 20 μL aliquots of SFMA hydrogel pre-gel solution at varying concentrations were precisely dispensed into each well of a 96-well plate (each concentration was replicated three times, *n* = 3). The solutions were then subjected to UV irradiation to facilitate crosslinking and solidification, forming stable hydrogel networks. To mimic physiological conditions, 100 μL of PBS buffer was added to each well. The transmittance of the SFMA hydrogels was subsequently evaluated using a microplate reader over a wavelength range of 400 to 800 nm. The Formula (1) for calculating the transmittance is as follows:

(1) $$Transmittance\;\left(T\right)=10^{-A}$$

Among them, *A* represents the absorbance of each group of hydrogels.

### 2.9. Mechanical Compression Testing of Microneedles

The mechanical properties of the microneedle were evaluated using a universal testing machine. The tip of the microneedle was mounted on the stainless-steel base of the testing machine. A vertical force was applied using a moving sensor at a controlled constant velocity of 0.05 mm/min, while simultaneously recording the force as a function of displacement.

### 2.10. Cytotoxicity and Cell Proliferation

The biocompatibility of microneedles was evaluated using human corneal epithelial cells (HCECs) and Mouse fibroblast cell line (L929). Live/Dead cell staining assay was employed. HCECs were cultivated in a specific medium, whereas L929 cells were maintained in low-glucose medium containing 5% fetal bovine serum (FBS) and 1% penicillin-streptomycin. All cultures were incubated at 37 °C and 5% CO_2_. Microneedle extract was prepared by incubating microneedles in the culture medium for 24 h, followed by filtration through a 0.22 µm membrane filter. The cytotoxicity and proliferation effects of microneedles on cells were assessed using this extract. First, a cell suspension of HCECs and L929 cells at a density of 2 × 10^4^ cells/well was prepared and seeded into a 24-well culture plate. The plate was then placed in a humidified incubator at 37 °C with 5% CO_2_ to allow cell attachment and proliferation under optimal conditions. After the cells adhered to the culture surface, the culture medium was replaced with the corresponding cell extract solution according to the experimental grouping, and further incubated for 24 h to enable the cells to interact with the extract. Subsequently, cell viability and death were assessed using a Calcein-AM/PI dual-color staining kit. Calcein-AM fluoresces green in viable cells due to its enzymatic conversion by esterases, whereas PI produces red fluorescence in apoptotic or necrotic cells by binding to their exposed DNA. To perform the staining, the culture medium was aspirated, and 500 µL of the staining solution was added to each well. The plate was incubated at 37 °C in the dark for 30 min to allow the dyes to interact with the cells. Finally, the fluorescence was observed and photographed under an inverted fluorescence microscope. In the acquired images, the green fluorescence indicated viable cells, while the red fluorescence marked apoptotic or necrotic cells. This visualization provided a clear and intuitive comparison of the effects of the different cell extracts on cell survival status.

Cell proliferation was quantitatively assessed using a Cell Counting Kit-8 (CCK-8, Bioss, Beijing, China). Cells were seeded in 96-well plates at 5 × 10^3^ cells per well (100 µL/well) and incubated overnight at 37 °C in 5% CO_2_. The next day, the medium was replaced with extract-containing medium. At 1, 4, and 7 days, 10 µL CCK-8 solution was added per well, incubated 30 min at 37 °C, and absorbance was measured at 450 nm using a microplate reader. Each experiment was triplicated and repeated thrice for reliability.

### 2.11. In Vitro Degradation

To evaluate the degradation performance of the SFMA and SF in microneedles, a 7-day degradation study was conducted. Initially, the dry weight of each microneedle (*M*_0_) was measured. The microneedles were then incubated in pH 7.4 PBS solution at 37 °C with continuous shaking at 200 rpm. At predetermined time intervals, the microneedles were removed from the solution, dried to remove moisture, and their remaining dry weight was measured (*M*_1_). The weight remaining of the microneedles was calculated using the following Formula (2):

(2) $$Weight\;remaining\left(\%\right)=\frac{M_{1}}{M_{0}}\times 100\%$$

### 2.12. Drug Releases Test

To simulate the encapsulation of proteins within microneedles, fluorescein isothiocyanate-labeled bovine serum albumin (FITC-BSA) was employed as a model. A mixed solution was prepared by blending 0.1 mL of FITC-BSA (at a concentration of 5 mg/mL) with 0.25 mL of SFMA pre-gel solution (with varying concentration gradients). An aliquot of 0.08 mL was withdrawn from this mixed solution and used to fabricate hydrogel spheres. Following sphere preparation, the spheres were immersed in 1 mL of PBS maintained at a constant temperature of 37 °C. Subsequently, 0.1 mL of the buffer was withdrawn for detection; to maintain a constant total volume in the system, an equal volume of PBS was supplemented to replace the withdrawn portion. This procedure was repeated three times for each concentration of the SFMA pre-gel solution. Finally, the collected samples were transferred to a 96-well plate, and their fluorescence intensity (with an excitation wavelength of 495 nm and an emission wavelength of 525 nm) was measured to establish a standard curve, which was used to calculate the release rate. The cumulative release of FITC-BSA was calculated using the following Formula (3) [33]:

(3) $$Cumulative\;FITC-BSA\;release\left(\%\right)=\frac{F_{t}{-F}_{0}}{F_{total}{-F}_{0}}\times 100\%$$

where *F_t_* is the fluorescence intensity at time t, *F*_0_ is the initial background fluorescence at time 0, and *F_total_* is the total fluorescence intensity after complete release of all encapsulated FITC-BSA.

### 2.13. Statistical Analysis

All results are expressed as the mean ± standard deviation (SD) based on at least three replicates. The Student’s *t*-test was used for data analysis between two groups, and one-way analysis of variance (ANOVA) was used for data analysis of three or more groups. Statistical significance was set as follows: * *p* < 0.05, ** *p* < 0.01, *** *p* < 0.001, **** *p* < 0.0001.

## 3. Results and Discussion

### 3.1. Preparation and Application of Microneedles

**Figure 1:**
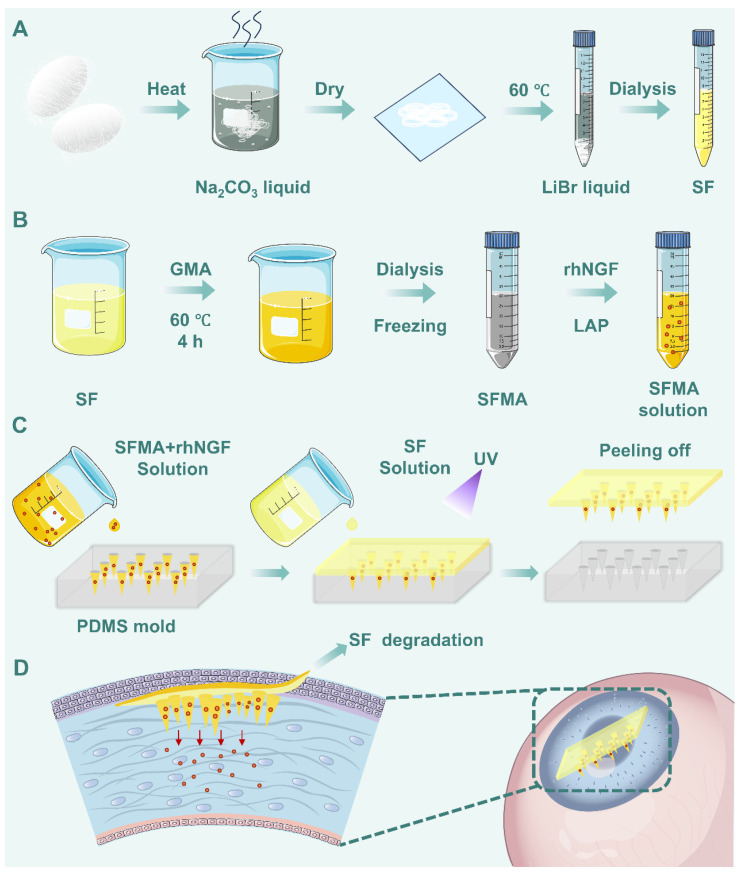
**Schematic illustration of the fabrication process for rhNGF-loaded SFMA microneedles.** (**A**) Preparation of the SF solution. (**B**) Fabrication of the SFMA hydrogel. (**C**) Fabrication of rhNGF-loaded SFMA microneedles. (**D**) Schematic diagram of the application of microneedles in corneal tissue repair.

SF solution was extracted from silkworm cocoons through degumming, drying, and dissolution (Figure 1A). Then, the SF solution was reacted with glycidyl methacrylate (GMA), followed by dialysis, freeze-drying, and mixing with Lithium phenyl-2,4,6-trimethylbenzoylphosphinate (LAP) solution to obtain the SFMA prepolymer (Figure 1B). For microneedle fabrication, SFMA was mixed with rhNGF and poured into a polydimethylsiloxane (PDMS) mold. After degassing in a vacuum chamber, excess SFMA/rhNGF solution was scraped off and recovered. UV crosslinking was performed, after which the bottom was filled with SF solution and dried overnight at 60 °C to complete the system (Figure 1C). The microneedle technology, due to its minimally invasive nature, precision, and sustainability, has demonstrated significant potential in the field of corneal tissue engineering (Figure 1D).

### 3.2. Synthesis and Characterization of SFMA

**Figure 2:**
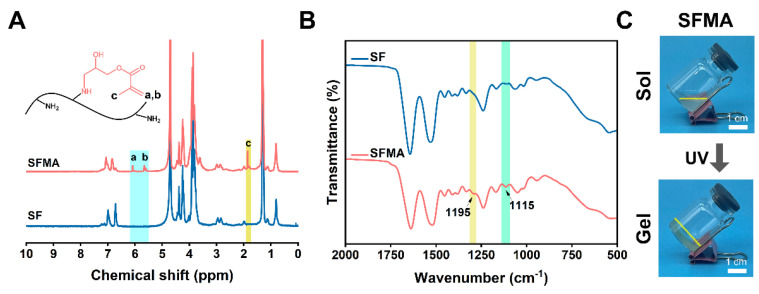
**Characterization of SFMA.** (**A**) ^1^H-NMR spectra of SFMA. (**B**) FTIR spectrum of SFMA hydrogel. (**C**) General view of SFMA hydrogel (sol–gel transition).

During SFMA synthesis, epoxy-amine click reactions were employed to couple the NH_2_ groups of lysine residues in the α-helical or β-sheet regions of SF with the reactive epoxy groups of GMA. This reaction successfully introduced GMA moieties onto the SF molecular structure. (Figure S1). The structure of SFMA was verified using ^1^H-NMR. Compared to the spectrum of SF, the SFMA spectrum exhibited characteristic signals from the GMA moieties: a signal at 1.8 ppm corresponding to the methyl group (c), and the vinyl group of methacrylate at 6.1–6.0 ppm (a) and 5.7–5.5 ppm (b) (Figure 2A). In the subsequent analysis, FTIR was employed to investigate the functional groups and secondary structure of SF and SFMA. Figure 2B presents a comparative analysis of the FTIR spectra of SF and SFMA, highlighting the spectral differences between the two. Notably, a new absorption peak at 1195 cm^−1^ emerges in the SFMA spectrum, which is characteristic of the C-O stretching vibration within the GMA group. Additionally, a distinct absorption at 1115 cm^−1^ is observed, corresponding to the CH bending vibration of the vinyl group. These spectral features provide compelling evidence for the successful integration of GMA moieties into the molecular architecture of SF. During the photo-crosslinking process, the SFMA hydrogel precursor solution was mixed with the LAP photoinitiator solution. Subsequent exposure to UV irradiation activated the LAP molecules to generate free radicals. These radicals initiated the polymerization of the vinyl groups in SFMA molecules, forming a three-dimensional network structure through a radical polymerization reaction (Figure S2). Figure 2C clearly illustrates the sol-to-gel phase transition of the SFMA hydrogel precursor upon UV irradiation, indicating the successful formation of a gel material as a result of the photo-crosslinking reaction.

### 3.3. Transmittance of the SFMA Hydrogel

**Figure 3:**
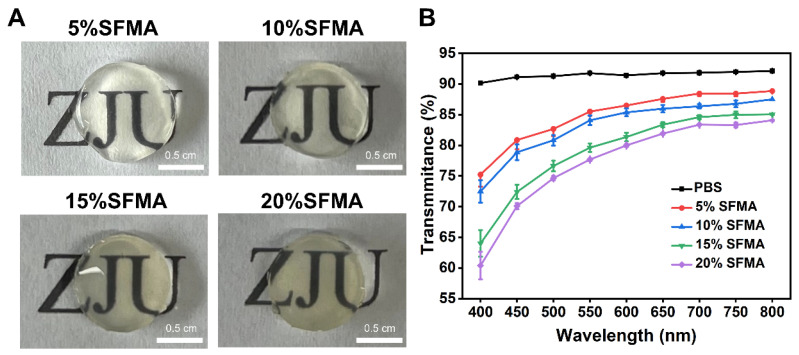
**Transmittance of the SFMA hydrogel.** (**A**) Photographs of different concentrations of SFM. Scale bars: 0.5 cm. (**B**) The average transmittance of different concentrations of SFMA (*n* = 3).

In corneal implant applications, the high transmittance of microneedles is of critical importance due to the direct impact of corneal optical transparency on light transmission and visual recovery. In this study, circular SFMA hydrogel specimens with a thickness of 2 mm were fabricated and coated onto the letter “ZJU”. The high optical transparency of the hydrogel was confirmed by the clear visibility of the letter (Figure 3A). The transmittance of SFMA hydrogels at various concentrations was evaluated. The results indicated that while the transmittance decreased with an increase in SFMA concentration, it remained above 60% even at the highest concentration tested, meeting the optical performance requirements for corneal implant materials (Figure 3B). This suggests that SFMA hydrogels possess excellent optical transparency alongside sufficient mechanical strength, highlighting their broad potential for application in corneal implants.

### 3.4. Preparation and Characterization of Microneedles

**Figure 4:**
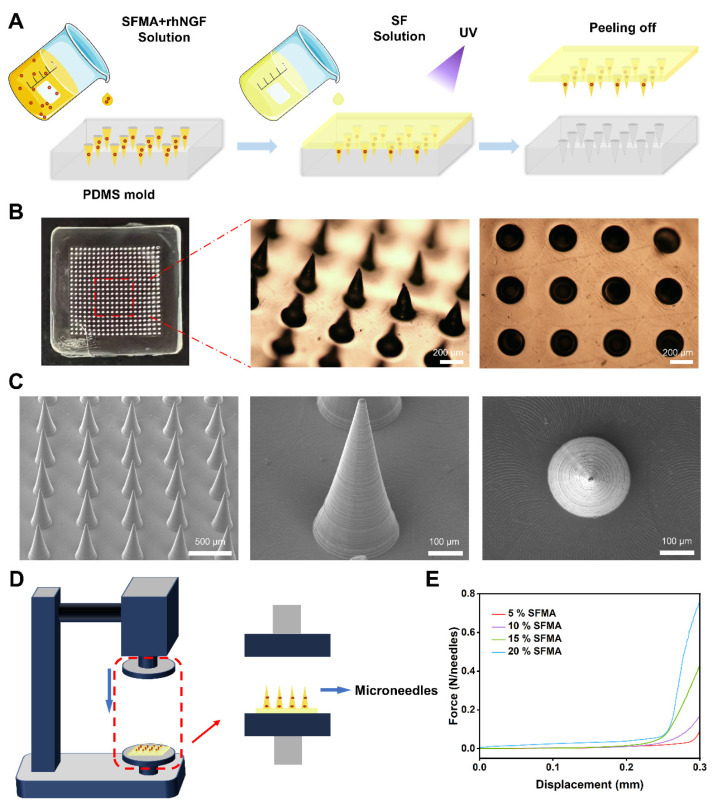
**Preparation and Characterization of Microneedles.** (**A**) The process of making detachable microneedles. (**B**) The microscopy images of the microneedles array. Scale bars: 200 µm. (**C**) SEM images of the SFMA microneedles. (**D**) Compression test schematic diagram. (**E**) The comparative mechanical strength assessment of SFMA microneedle.

In the fabrication of microneedles, SFMA was selected as the primary material for the microneedle tips, while SF solutions (6.5% concentration) were employed for the backing layer. This choice was due to SF’s excellent biocompatibility, degradability, high transparency, and high mechanical strength [34,35]. The fabrication process was divided into two main steps. First, SFMA and rhNGF mixed solutions were precisely filled into the tip regions of PDMS molds using a vacuum device. Excess solution was carefully removed using a scraper, and the tip structures were fixed via photopolymerization. Subsequently, the backing layer was formed by covering the tip with a 6.5% SF solution. After drying overnight at 30 °C, the molds were removed to obtain intact microneedle patches (Figure 4A). The microneedles were observed under a stereoscopic microscope, forming a regular 20 × 20 array with a tip-to-tip distance of 550 µm and a height of 600 µm (Figure 4B). Scanning electron microscopy (SEM) imaging revealed the clear conical tip structure (Figure 4C). This conical design, characterized by its sharp tip, ensures minimal deformation and high mechanical strength, thereby enhancing corneal penetration efficiency and stability. Additionally, the design effectively reduces tissue insertion resistance while concentrating the drug at the tip to increase local drug concentration. Furthermore, the reduced tissue damage makes microneedles more suitable for ophthalmic applications.

Compression tests were conducted to evaluate the mechanical properties of microneedles with varying SFMA concentrations. Figure 4D presents a schematic of the compression test, showing the testing procedure used for the quantitative analysis of mechanical properties under compression, which ensures sufficient strength and stability for corneal puncture applications. By fabricating microneedles with varying concentrations of SFMA and conducting precise mechanical tests, we found that the mechanical strength of the microneedles increased significantly with higher SFMA concentrations. During the experiments, when the sensor contacted the microneedle tip and applied force until a displacement of 0.3 mm, the microneedles demonstrated sufficient mechanical strength to withstand nearly 0.1 N of force (Figure 4E). We have systematically compared the fracture force of our microneedles (~0.1 N) with literature-reported force thresholds for corneal epithelium penetration. Zhang et al. reported a penetration force of approximately 0.1 N, while Yu et al. demonstrated that this threshold could be as low as 0.04 N [36,37]. This ensures effective penetration of the corneal epithelium.

### 3.5. Cytotoxicity and Cell Proliferation

HCECs play a vital role in corneal tissue engineering by functioning as a major component of the corneal epithelial barrier. Under normal physiological conditions, HCECs collaborate with other corneal tissues to form a robust physical barrier and immune defense system, protecting against microbial invasion and foreign object penetration. Their tightly packed arrangement enhances the structural integrity of the corneal epithelium, while the coordinated action of aquaporins ensures precise regulation of corneal hydration. By maintaining the dehydrated state and transparency of the cornea, HCECs ensure optimal light transmission and provide the foundation for clear vision. L929 cells, as a widely used model system, play a crucial role in evaluating the applicability of materials for corneal tissue engineering. Analysis of their growth, adhesion, proliferation, and metabolic activity on the material surface offers essential preliminary insights into material suitability. Consequently, in this study, both HCECs and L929 cells were utilized to assess the biocompatibility of the materials.

**Figure 5:**
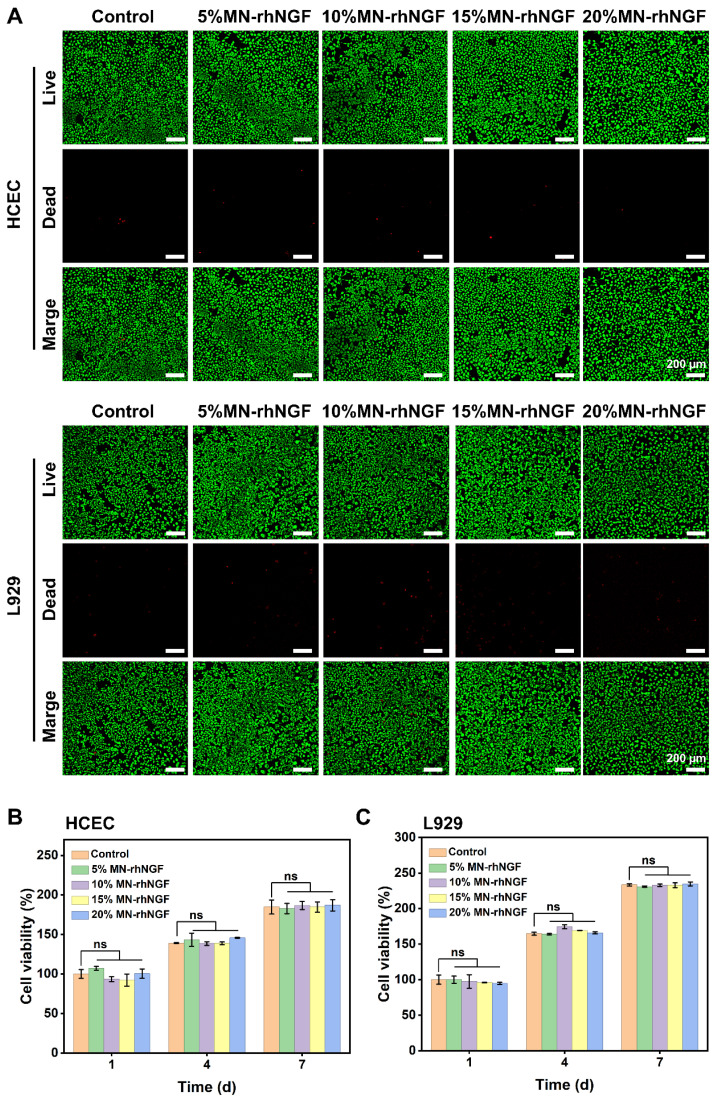
**Cell biocompatibility and proliferation.** (**A**) Evaluate the live/dead cells of HCECs and L929 after co-culture with different concentrations of MN-rhNGF microneedle extract for 24 h. Scale bars: 200 µm. (**B**,**C**) Proliferation of HCECs/L929 cells cultured with extract of MN-rhNGF microneedle. Data are presented as mean ± SD (*n* = 3). ns stands for not significant.

Cytotoxicity is a pivotal factor in implantable material research. This study systematically evaluated the cytocompatibility of MN-rhNGF hydrogels at concentrations of 5%, 10%, 15%, and 20% using live/dead staining and CCK-8 assays on HCECs and L929 cell lines. The control group consisted of blank controls without material addition. Fluorescence micrographs revealed green fluorescent viable cells and red apoptotic/dead cells in HCEC and L929 cultures exposed to hydrogel extracts. No significant increase in apoptosis or cell death was observed in any concentration group compared to the control, indicating low cytotoxicity of MN-rhNGF (Figure 5A). CCK-8 assay results further confirmed these observations. Cell viability was measured on days 1, 4, and 7, with the absorbance of the control group on day 1 normalized to 100%. Throughout the culture period, no statistically significant difference in cell viability was found between any MN-rhNGF concentration group and the control group for either HCECs or L929 cells (*p* > 0.05) (Figure 5B,C). In summary, MN-rhNGF hydrogels demonstrated excellent cytocompatibility across the tested concentration range, with no significant cytotoxicity observed.

### 3.6. Degradation and Sustained Release

**Figure 6:**
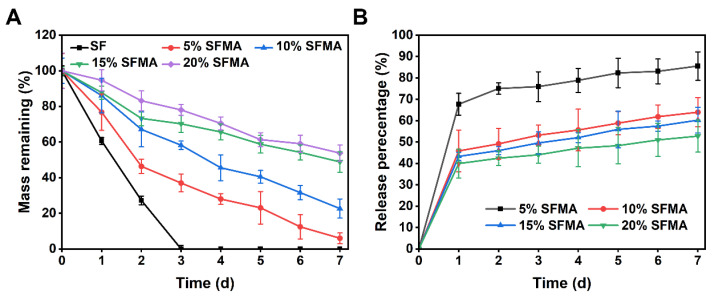
**Degradation and sustained release.** (**A**) In vitro degradation rates of SF and SFMA hydrogels with different concentrations. (**B**) The drug release test in different concentrations of SFMA.

Figure 6A systematically characterizes the in vitro degradation kinetic profiles of SF and SFMA hydrogels with varying glycidyl methacrylate substitution degrees in phosphate-buffered saline (PBS, pH 7.4). As shown, the unmodified SF film exhibited rapid degradation kinetics, with its mass completely depleted within 3 days, attributable to its high water solubility. In contrast, SFMA hydrogels displayed a significantly delayed and highly linear mass loss behavior, where the degradation rate was inversely correlated with the degree of glycidyl methacrylate substitution. Specifically, the low-substitution SFMA (5%) lost approximately 95% of its mass within 7 days, whereas the high-substitution group (20%) degraded by only ~50% during the same period. Corneal nerve repair requires stable drug delivery for at least two weeks. High-concentration SFMA hydrogels theoretically possess the feasibility to match the therapeutic duration, ensuring sustained effective drug concentrations throughout the critical period of nerve regeneration.

To simulate the delivery behavior of nerve growth factor from microneedles, this study employed FITC-BSA as a model drug to systematically investigate its in vitro release kinetics. Rieber et al. demonstrated that FITC-BSA (66 kDa) and insulin-like growth factor-1 (IGF-1, 7.6 kDa) exhibit identical release kinetic profiles in collagen scaffolds [38]. Yao et al. similarly employed FITC-BSA as a model for large-molecular-weight protein drugs to systematically evaluate in vitro protein release from hydrogels [9]. Furthermore, recent studies have shown that diffusion coefficients for therapeutic proteins within the 10–100 kDa range typically vary by less than 2-fold, further supporting the rationale for our model protein selection [39]. Specifically, 0.1 mL of FITC-BSA was incorporated into SFMA pre-gels of varying concentrations, followed by photo-crosslinking and subsequent immersion in PBS. The cumulative release over a 7 days period was quantified using the fluorescence detection module of a microplate reader. The results demonstrated that the drug-loaded SFMA hydrogels exhibited a characteristic biphasic release profile, comprising an initial burst release phase within the first 24 h, followed by a sustained, linear slow-release phase from days 2 to 7. This release mechanism can be attributed to two release processes: the first release stage diffusion-mediated burst release of surface-associated drug molecules through the hydrogel network, and the second release stage sustained release of the encapsulated payload concurrent with hydrogel matrix degradation (Figure 6B). Given that clinical corneal nerve regeneration requires sustained drug exposure for a minimum of two weeks, this sustained-release system can effectively fulfill the demand for prolonged drug delivery, significantly reduce dosing frequency, and thereby enhance patient compliance.

### 3.7. Limitations and Future Work

The present study primarily focused on evaluating the therapeutic feasibility. As such, direct in vitro investigations targeting neuronal and neuron-supporting cells were not included, and systematic in vivo mechanistic analyses were beyond the scope of the current work. Consequently, the molecular pathways underlying the observed nerve regenerative effects were not fully elucidated in this study.

To address these limitations and further strengthen the mechanistic and translational understanding of this system, several follow-up studies are planned. First, systematic in vitro mechanistic investigations using neuronal and neuron-supporting cell models will be conducted to clarify the molecular pathways involved in nerve regeneration. Second, extended safety evaluations, including 28-day cytotoxicity testing and corneal endothelial cell-specific responses (such as inflammatory factor profiling), will be incorporated into subsequent preclinical studies following final material formulation optimization. Finally, detailed biodistribution and pharmacokinetic analyses will be performed as part of future preclinical investigations to support further translational development.

## 4. Conclusions

In this study, we developed an rhNGF-loaded microneedle MN drug delivery system, offering a highly promising therapeutic strategy for corneal tissue repair. Compared to conventional eye drops and other rhNGF delivery modalities, which typically suffer from bioavailability of less than 5% and are severely constrained by tear clearance, corneal epithelial barrier, and other physiological factors, our system precisely encapsulates rhNGF within the microneedle tips. Upon application, the microneedles penetrate the corneal epithelium, enabling direct drug deposition into the subepithelial stromal region, thereby facilitating targeted delivery and demonstrating potential for enhanced drug absorption. In vitro cytocompatibility assessments confirmed that the hydrogel material exhibits no significant cytotoxicity toward HCECs and L929, demonstrating excellent ocular biosafety. In summary, this rhNGF-loaded microneedle patch, serving as an efficient and minimally invasive ocular drug delivery vehicle, establishes a novel technological pathway for promoting functional reconstruction of corneal tissue.

## Data Availability

The original contributions presented in this study are included in the article/Supplementary Material. Further inquiries can be directed to the corresponding author.

## Supplementary Materials

The following supporting information can be downloaded at: https://www.mdpi.com/article/10.3390/polym18030412/s1, Figure S1: The synthesis mechanism of SFMA; Figure S2: The gelation mechanism of SFMA.
