# Supplementary figures and images for: Silk Fibroin Hydrogel Microneedles Loaded with Recombinant Human Nerve Growth Factor for Corneal Tissue Engineering
Source: Polymers (Basel). 2026 Feb 5;18(3):412. doi: 10.3390/polym18030412 (PMC12899253; doi:10.3390/polym18030412)

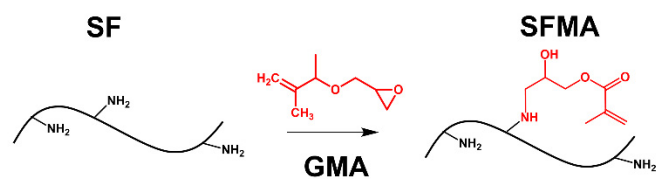

**Figure S1. The synthesis mechanism of SFMA.**

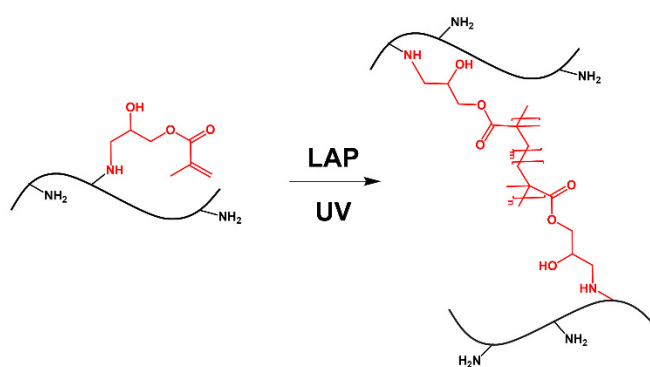

**Figure S2. The gelation mechanism of SFMA.**

Supplement: Supplementary file 1 [file polymers-18-00412-s001.zip › polymers-4114132-supplementary.pdf]
